# The Concluding Part of a Correspondence Which Appeared in the Medical and Physical Journal of Last Month

**Published:** 1820-04

**Authors:** 


FOR THE LONDON MEDICAL AND PHYSICAL JOURNAL.

*The concluding part of a Correspondence which appeared in the Medical and Physical Journal of last month.*

ABOUT a fortnight after the date of Mr. Brodie's last letter, Dr. W. Philip addressed the following to that gentleman.

*Worcester, March 3d,* 1820.

SIR,—As I have received no answer to my first query, relating to the manner in which the animal was prepared for your experiment, and this appears to me of considerable consequence in settling the point between us, I will thank you for your answer; and also to inform me whether there was any appearance of inflammation in the stomach of either of the rabbits.

I have the honour to be, Sir, your faithful humble servant,

A. P. W. PHILIP.

*To B. C. Brodie, Esq.*

Mr. Brodie returned the following answer.

16, *Saville Row; March* 5*th,* 1820.

Sir,—Both animals in my last experiment were fed after a fast of about sixteen hours. I did not observe any appearance of inflammation in the stomach of either of them, but my attention was not particularly directed to this point.

I have the honour to be, Sir, your obedient servant,

B. C. BRODIE.

*To Dr. Wilson Philip.*

The following is Dr. W. Philip's reply to the above.

*Worcester; March* 9*th,* 1820.

Sir,—I yesterday received your reply to my last letter, from which it appears, that the animals were properly prepared for the experiment. Your answer to my question respecting the appearance of inflammation greatly surprises me, as, by referring to my Inquiry, you will see that I have from the first regarded inflammation of the stomach as the criterion that the proper power of galvanism had been employed. In the 216th page of the first edition it is said, " We could never succeed ;" that is, in the newly dead animal, " in producing the slightest appearance of inflammation, either in the stomach or bowels, *an effect which uniformly attends digestion supported by galvanism.*" And again, page 135, " the thoracic viscera, in short, were rather in a state of high inflammation, *an effect always produced by a considerable galvanic power in the part to which it is directed.*" I therefore expected, that, in repeating my experiment, your attention would have been particularly directed to this circumstance. It is impossible however to see a stomach, which has for a sufficient length of time been exposed to such a power of galvanism as I have found necessary to produce digestion, without being struck with its inflamed appearance.*

I mentioned in my letter of the 14th ult. such reasons as assured me, that the stomach, in your last experiment, had not been sufficiently exposed to the galvanic influence. What you now state, leaves no doubt on this head. As there was little or no inflammation excited in the stomach, it is *certain*, that the galvanic power which you employed, was either too weak, or not sufficiently directed to the stomach. We need look no further for the difference of result between your experiment and mine. Thus it appears, that both your repetitions of my experiment leave the subject in precisely the same predicament as if neither of them had been made, with this exception, that the

* The inflammation of the organ to which the galvanism is chiefly directed, is easily accounted for. It is not to be supposed, that we can by the artificial application of galvanism, direct it in the proper quantity to the proper parts in such a way, as not to supply more to some and less to others, than nature supplies.

efforts to vomit having been prevented by so small a power of galvanism, affords a striking, because an unintentional, confirmation of the result I obtained.

In case the experiment should again be repeated, I think it necessary to call your attention to the following circumstances, which are not less essential, than the degree of the galvanism and its mode of application. Although your experiments stand in opposition only to my shorter galvanic experiment on rabbits, for as to that in which the galvanism was continued for sixteen hours, no attempt has been made to repeat it, yet you adopt the rule which, according to my method, necessarily produces an experiment of long continuance; for your object is to employ no stronger power of galvanism than is requisite to produce a twitching in the fore legs. In my shorter experiment, which lasted only six hours, the power of the galvanism was much beyond this degree, producing general contraction of the muscles, (page 224, 2d edit.) and proving fatal to the animal in the above short space of time. I found it more conclusive to employ a comparatively weak power of galvanism, that the animal might be longer exposed to it; but you employed this weak power, and yet, by killing the animal at the end of five or seven hours, defeated my object in having recourse to it. You wholly deviated from my principle, when you killed the animal by any other means than the galvanism. You will perceive, from all I have had occasion to say, how very far from correct is the observation in your first letter,—that you could discover only one point of difference between your first experiment and mine.

It is also proper to remark, that the old trough, which I observe in my treatise I always used, is preferable in such experiments to the improved pile, because in the former, the number of plates is greater in proportion to the surface, and I have found, that it is on the intensity, not on the quantity, of the electric power, that the above effects on the animal body depend. I had occasion to address some observations on this subject to Dr. Thomson, which were published in the eleventh volume of the Annals of Philosophy, page 117.*

I have the honour to be, Sir, your faithful humble servant,

A. P. W. PHILIP.

*To B. C. Brodie, Esq.*

---

* Although I have in my first letter replied to an experiment of Mr. Brodie's on the frog, I think it proper to observe, in concluding this correspondence, that had the wound healed perfectly in this experiment, it could not have been adduced as an argument against inferences from experiments made on the warm-blooded animal; for the functions of the nervous system in warm and cold blooded animals differ so much, that the frog will perform motions of volition, and even sit in its usual posture, for several days after the head is removed. When we descend lower in the scale, we find animals capable of regenerating whole numbers; and at length some, the different parts of which, when they are cut into pieces, live as distinct animals.

